# Vaccinia Virus as a Master of Host Shutoff Induction: Targeting Processes of the Central Dogma and Beyond

**DOI:** 10.3390/pathogens9050400

**Published:** 2020-05-21

**Authors:** Pragyesh Dhungel, Fernando M. Cantu, Joshua A. Molina, Zhilong Yang

**Affiliations:** Division of Biology, Kansas State University, Manhattan, KS 66506, USA; fcantu@ksu.edu (F.M.C.); molina2388@ksu.edu (J.A.M.)

**Keywords:** poxviruses, vaccinia virus, virus–host interactions, host shutoff, transcription, mRNA processing, translation, central dogma

## Abstract

The synthesis of host cell proteins is adversely inhibited in many virus infections, whereas viral proteins are efficiently synthesized. This phenomenon leads to the accumulation of viral proteins concurrently with a profound decline in global host protein synthesis, a phenomenon often termed “host shutoff”. To induce host shutoff, a virus may target various steps of gene expression, as well as pre- and post-gene expression processes. During infection, vaccinia virus (VACV), the prototype poxvirus, targets all major processes of the central dogma of genetics, as well as pre-transcription and post-translation steps to hinder host cell protein production. In this article, we review the strategies used by VACV to induce host shutoff in the context of strategies employed by other viruses. We elaborate on how VACV induces host shutoff by targeting host cell DNA synthesis, RNA production and processing, mRNA translation, and protein degradation. We emphasize the topics on VACV’s approaches toward modulating mRNA processing, stability, and translation during infection. Finally, we propose avenues for future investigations, which will facilitate our understanding of poxvirus biology, as well as fundamental cellular gene expression and regulation mechanisms.

## 1. Introduction to Host Shutoff

Viruses are obligatory intracellular parasites that only replicate in host cells. Although viruses encode various numbers of genes to perform replication, they rely on cellular translation machinery for protein synthesis. In other words, competition exists between viruses and their hosts to utilize the limited amount of cellular translation machinery. This situation instigates many viruses to induce a rapid and profound decline in global host protein synthesis while continuously synthesizing the viral proteins, a phenomenon often termed “host shutoff”. Viruses encode proteins that lead to host shutoff by affecting many cellular gene expression processes. Studies on virus-induced host shutoff to date have indicated that some viruses allocate proteins to inhibit DNA synthesis and transcription; more often, many viruses encode an arsenal of proteins targeting mRNA processing and translation. One of the most energy-consuming processes in the cell is mRNA translation [1,2]. By usurping host mRNA translation, viruses can gain a competitive advantage over available energy and translational machinery, thereby enhancing the expression of viral mRNA to protein. Recently, it has also been evident that viruses can limit host cell proteins post-translationally by inducing rapid protein degradation [3,4]. Because the targeted proteins include newly synthesized proteins, such as antiviral proteins, it also contributes to host shutoff. 

Upon viral infection, the host cell produces an innate immune response as a front line of defense, which primes the adaptive immune response to clear viral infection at the organismal level. Despite the host cells having an efficient innate and adaptive immunity-mediated antiviral response, viruses have evolved diverse strategies to impede, modulate, and evade antiviral responses. In addition to encoding multiple classes of immunomodulatory proteins, the abolishment of the host cells’ ability to produce proteins, including those involved in an antiviral response, provides viruses with a powerful strategy to counteract an immune response. 

Host shutoff must occur within a certain time frame of the virus replication cycle; for many viruses, this could merely be a few hours during the early time of infection. Within a short time, a virus must use one or more tactics to induce a quick and continuous host shutoff and divert cellular resources toward the synthesis of viral macromolecules. Here, we briefly review the strategies used by various viruses to induce host shutoff. Vaccinia virus (VACV), the prototypic member of poxviruses, turns out to be a master at inducing host shutoff through targeting the genetic information flow of the central dogma, from transcription to translation, as well as pre-transcription and post-translation processes. We discuss VACV-induced host shutoff in detail in this review. Still, numerous questions remain unanswered regarding understanding VACV-induced host shutoff, which provides many opportunities to elucidate mechanisms of poxvirus replication and fundamental cellular biology mechanisms. 

## 2. Vaccinia Virus 

Historically, poxviruses have been responsible for many important human and animal diseases, including smallpox, molluscum contagiosum, monkeypox, cowpox, goatpox, lumpy skin disease, and sore mouth infection [5]. Smallpox, caused by the variola virus (VARV), was one of the deadliest diseases in human history and was eradicated in the mid-late twentieth century after a global vaccination campaign using another member of the *Poxviridae* family, VACV [6,7,8]. Poxviruses are useful as vaccine vectors against other viruses, such as human immunodeficiency virus (HIV), severe acute respiratory syndrome coronavirus (SARS-CoV), Rabies virus, and Zika virus, among others [9,10,11,12,13]. Numerous reviews have comprehensively summarized the range of VACV-based vaccines [14,15]. Additionally, some poxviruses have been developed to be as oncolytic virotherapy agents, which can also be used in combination with other therapies to increase desirable patient outcomes, as is nicely outlined in multiple reviews [16,17].

Besides being used to eliminate smallpox, VACV is one of the most extensively studied poxviruses. Similar to all other poxviruses, VACV has a linear, non-segmented, double-stranded DNA genome, and replicates in the cytoplasm [5]. The VACV genome is approximately 190 kilobase pairs (kbp) in length with a hairpin loop at each terminus [18]. To sustain cytosolic replication, transmission between hosts, and the evasion of the host immune system, over 200 proteins are encoded by the VACV genome. In addition to the structural proteins used to make the viral particle, the genome also encodes enzymes responsible for viral DNA replication, transcription, mRNA modification—such as capping, 2′O methylation, decapping, and polyadenylation—at least 15 proteins for entry (4 for binding and 11 for fusion), and a plethora of immunomodulatory enzymes alongside protein kinases and proteases [5,19]. Expression of the VACV genome occurs in a temporally regulated cascade fashion distinguished as early, intermediate, and late genes. Early VACV genes are comprised of 118 open reading frames (ORFs) that are expressed before viral DNA replication, while the 93 intermediate and late genes are expressed after viral DNA replication [20,21]. Some genes at each stage can continue to be transcriptionally active throughout infection via multistage promoters [22]. 

## 3. Cellular DNA Synthesis Inhibition 

DNA is the cellular genetic material encoding information that proceeds to transcripts and proteins. Some viruses have evolved means to modulate host DNA. Kit et al. showed that poxvirus infection induces a rapid decrease in host cell DNA synthesis [23,24,25]. To decrease host cell DNA synthesis, VACV infection suppresses host nuclear DNA polymerase activity as early as 2 h post-infection. The studies measuring ^14^C- or ^3^H-labeled thymidine incorporation into the host and viral DNA during VACV infection observed an immediate decrease in labeled thymidine incorporation in host DNA. In contrast, labeled thymidine incorporation in viral DNA increased until 3 h post-infection, corresponding to the time when viral DNA replication occurs [26,27]. Heat-inactivated and UV-irradiated non-infectious VACV could inhibit host DNA synthesis, indicating that protein(s) in the virion are responsible for the inhibition [27]. However, the factors are not yet identified. 

**Figure 1:**
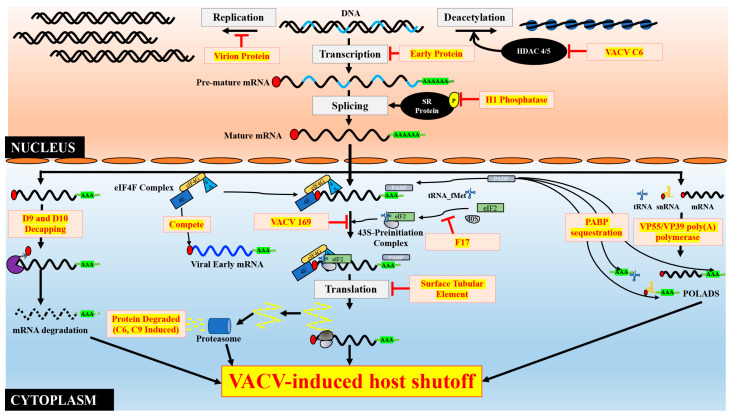
Summary of vaccinia virus (VACV)-induced host protein synthesis shutoff. VACV targets all the macromolecules in the central dogma of genetics, i.e., DNA, RNA, and proteins. VACV inhibits DNA replication (virion proteins), inhibits transcription (early protein(s)), interferes with mRNA processing, such as polyadenylation (VP55/VP39) and splicing (H1 phosphatase), and induces mRNA degradation (D9/D10). Further, VACV hinders 43S preinitiation complex formation (F17), decreases polysome bound mRNAs (surface tubular element), and inhibits translation (VACV 169). Additionally, VACV infection leads to the redistribution and post-translational modifications of translation initiation factors, which confers a translational advantage to viral mRNAs and a disadvantage to cellular mRNAs. Furthermore, VACV infection accelerates cellular protein degradation, including newly synthesized proteins. Abbreviations: HDAC 4/5, histone deacetylase 4/5; SR protein, serine (S)/arginine (R)-rich protein; eIF, eukaryotic translation initiation factor; eIF4A and eIF4E are abbreviated to 4A and 4E, respectively; tRNA, transfer RNA; tRNA_fMet, initiator-methionine tRNA; mRNA, messenger RNA; snRNA, small non-coding RNA; POLADS, polyadenylated short sequences; PABP, poly(A) binding protein; 40S, eukaryotic small ribosomal subunit.

Some other viruses also decrease host DNA synthesis or even degrade the host cell DNA. Notable examples include the virion-associated protein of frog virus 3 (FV3), S1 gene of reovirus, ICP10 of herpes simplex virus type 2 (HSV-2), and a small segment at the 3′ end of the vesicular stomatitis virus (VSV) genome inhibits host DNA synthesis via largely unknown mechanisms [28,29,30,31]. Bacteriophage T4D-induced exo- and endodeoxynucleases degrade bacterial host DNA within 5 minutes post-infection [32]. Because DNA is the source of genetic information used to synthesize host proteins, a decrease in host cell DNA likely contributes to host shutoff induction. However, the effect will need time to exert and it is unlikely to be the primary mechanism used to induce a rapid host protein synthesis shutoff (Figure 1).

## 4. Prevention of Cellular RNA Synthesis 

Different classes of RNAs are transcribed by three DNA-dependent RNA polymerase enzymes in eukaryotic cells. Transcription by RNA polymerase I (RNAPI) synthesizes ribosomal RNAs (rRNAs). RNA polymerase II (RNAPII) yields messenger RNAs (mRNAs), and RNA polymerase III (RNAPIII) produces transfer RNAs (tRNAs), small non-coding RNAs (snRNAs), micro RNAs (miRNAs), 5S RNA (5S), and small nucleolar RNAs (snoRNAs) [33,34]. The prevention of cellular mRNA synthesis provides viral mRNAs with the advantage of accessing cellular translation machinery with fewer competitors. Many viruses target RNAPII enzymes to inhibit cellular mRNA synthesis, while other viruses use their RNA polymerases for viral mRNA transcription. In two previous studies, the rate of RNA synthesis was determined using ^3^H- and ^14^C-uridine uptake experiments. These experiments demonstrated that a 60% and 90% reduction in uridine uptake during VACV infection occurred at 3 and 9 h post-infection, respectively, compared to the uninfected condition [23,35]. Unlike the inhibition of DNA polymerase activity, infectious VACV and the expression of early viral genes are required to inhibit RNAPII activity [35]. Again, the mechanism of VACV-induced host transcription silencing remains unanswered, and further studies are needed to elucidate the viral and cellular mechanisms involved in this process. Interestingly, Teferi et al. showed that the viral K7 protein promotes histone methylation associated with heterochromatin formation, suggesting epigenetic modulation is involved in this process [36]. Other epigenetic and genetic mechanisms may also lead to VACV-induced transcription silencing.Additionally, VACV infection induces a global degradation of host and viral mRNA, which is discussed in more detail in a later section.

VACV is not alone in the transcriptional silencing of host cells. Foot-and-mouth disease virus (FMDV) encodes 3C protease that cleaves histone H3, thus affecting the regulatory domain necessary for transcription [37]. Influenza A virus (IAV) uses multiple strategies to diminish host transcription. In the early stages of infection, IAV inhibits RNAPII elongation, followed by RNAPII degradation in the later stages. IAV infection also induces stress that leads to the failure of RNAPII termination at the polyadenylation signal (PAS), which interferes with host mRNA maturation [38,39,40]. A recent study indicated that the deregulation of RNAPII is dependent on the viral NS1 protein and can be augmented by the post-translational SUMOylation of an intrinsically disordered region of the IAV NS1 protein [41]. Like IAV, herpes simplex virus type 1 (HSV-1) also induces the failure of RNAPII to terminate at PAS likely through the binding between the immediate early protein ICP27 and 3′ processing factor CPSF to prevent the 3′ cleavage of mRNA [42,43]. Additionally, HSV-1 infection decreases RNAPII occupancy in two-thirds of cellular genes [44]. In fact, HSV-1 proteins ICP4 and ICP22 are known to dysregulate RNAPII-mediated transcription initiation and elongation [45,46,47]. 

## 5. Targeting mRNA Processing 

Messenger RNA that codes for a protein is synthesized as pre-mRNA. Pre-mRNA undergoes multiple processing steps to form mature mRNA. VACV encodes proteins that modulate many of these steps, including capping, polyadenylation, splicing, and the export of mRNA from the nucleus to the cytoplasm.

### 5.1. Capping

During transcription, when the length of cellular mRNA reaches 25–30 nucleotides, 7-methylguanosine cap (m^7^G) is added to the 5′ end of the transcript, which not only protects the mRNA from 5′-3′ exoribonucleases, such as Xrn1, it also helps the mRNA to be efficiently translated through the cap-dependent initiation mode. VACV mRNA capping is carried out in three reactions performed by viral enzymes. In this process, the newly synthesized transcript with a 5′ triphosphate is cleaved to produce diphosphate by RNA triphosphatase. Guanosine monophosphate (GMP) is then added by RNA guanylyl-transferase and subsequently methylated at the N7 position by RNA (guanine-N7) methyltransferase. VACV encodes a heterodimeric capping enzyme complex consisting of D1 and D12 proteins [48,49]. Additionally, VACV encodes the VP39 protein (J3R), which adds a methyl group at the 2′-O position of the first transcribed nucleotide (cap 1) adjacent to the 5′ cap (cap 0). This modification is present in higher eukaryotic mRNAs. However, apart from poxviruses, some other viruses, including HIV-1 (by host FTSJ3 protein), coronaviruses (by NSP16), and flaviviruses (by NS5 protein), have also been reported to contain 2′-O methylation in cap 1 [50,51,52,53,54]. The host’s innate immune system recognizes hypomethylated viral RNA via melanoma differentiation-associated protein 5 (MDA5), retinoic acid-inducible gene I (RIG-1), and interferon-induced proteins with tetratricopeptide repeats 1 (IFIT-1) to distinguish between self and non-self mRNAs, thereby preventing non-self mRNA translation [55,56,57]. Through methylation at the 2′-O position of cap 1, VACV may evade the host immune response and sustain its mRNA translation [53]. Another strategy that VACV uses is to remove the 5′ cap of mRNA using two decapping enzymes (D9 and D10) (Figure 1) [58,59]. The decapped mRNAs are then degraded by 5′-3′ exonucleases, such as Xrn1 [60]. The decapping process leading to mRNA degradation is discussed further in the mRNA degradation section.

Many viruses have developed strategies to remove the 5′ cap to induce mRNA degradation. The influenza virus cleaves cellular mRNA 5′ caps and uses the cleaved oligonucleotide as a primer for its transcripts through a mechanism known as “cap snatching” [61]. To perform cap snatching, polymerase basic protein 2 (PB-2) binds to the host mRNA cap through an extensive conformational change, followed by cleavage of the cap from host mRNA using the endonuclease in polymerase acidic protein (PA). PB-1 uses the cleaved cap as a primer for viral mRNA transcription [62]. Hantavirus also performs cap snatching using its N protein, which binds to the cellular mRNA cap and rescues capped mRNA fragments from the processing-bodies’ post-mRNA degradation. The capped mRNA fragment is further processed using viral RNA-dependent RNA polymerase, which is used as the primer for viral mRNA synthesis [63]. Recently, the African swine fever virus (ASFV) was shown to encode a decapping protein, ASFV-DP, which removes the m^7^G cap from both the host and viral mRNAs [64]. However, the physiological role of the decapping enzyme in ASFV infection is not elucidated yet.

### 5.2. Polyadenylation

Polyadenylation is the process of adding a stretch of adenine bases at the 3′ end of mRNA. It is an essential process for mRNA maturation that is tightly coupled with transcription termination. Polyadenylation protects mRNA from 3′-5′ exoribonucleases and assists in the optimal translation of mRNAs [65]. To protect viral mRNA and allow for optimal translation, VACV encodes a heterodimeric poly(A) polymerase complex of VP55/VP39 proteins early during infection that adds a poly(A) tail at the 3′ end of viral mRNAs [66,67,68]. The VP55 (E1L) protein functions as a catalytic component, whereas VP39 (J3R) protein acts as a processivity factor. During VACV infection, apart from catalyzing polyadenylation in viral mRNA VACV poly(A), viral polymerase polyadenylates cellular RNAs, such as tRNA, small nuclear RNA (snRNA), and mRNA, as well as small mRNAs that originated from the VACV genome to form non-translating polyadenylated short sequences (POLADS) [69]. The POLADs may affect host mRNA translation that will be discussed in detail in a later section. A separate interesting finding is that VACV VP55 protein polyadenylates host miRNAs, thereby leading to miRNA degradation. While the degradation of these miRNAs may abolish their effects on cellular and viral mRNAs, it is not clear how this mechanism may selectively affect host protein synthesis [70] (Figure 1). 

Other viruses have evolved means to affect mRNA stability by targeting the polyadenylation process. Nonstructural protein 1 (NS1a) of IAV restricts the polyadenylation of the nascent host mRNA by interacting with cleavage-polyadenylation specificity factor 30 (CPSF30). CPSFs are responsible for recognizing a polyadenylation signal in pre-mRNA and cleave the pre-mRNA, followed by the polyadenylation of upstream cleaved products [71]. In contrast to IAV’s strategy of blocking polyadenylation, Kaposi’s sarcoma-associated herpesvirus (KSHV) hyper-adenylates host transcripts in the nucleus after nucleolar translocation of PABP due to the SOX-protein-induced reduction of cytoplasmic mRNA, which leads to nuclear retention and the decreased stability of transcripts [72,73,74,75].

### 5.3. Splicing 

A pre-mRNA contains coding exons and noncoding introns. During the maturation of pre-mRNA, the introns are removed, and exons are joined through a process called splicing. VACV genes lack introns; hence, splicing is dispensable for VACV. However, VACV can target host-splicing machinery to induce host shutoff. The serine (S)/arginine (R)-rich protein (SR protein) family is an important protein family that is required for spliceosome assembly [76]. SR proteins are hyper-phosphorylated at a serine residue that is required for its function [77]. VACV-encoded protein phosphatase H1 may inactivate SR protein, thereby dysregulating the host cell’s RNA splicing machinery. The incubation of VACV H1 with SR protein from HeLa cell lysate de-phosphorylates SR protein, while the mechanism in VACV-infected cells has not been elucidated [78]. Since splicing is not required for VACV, it likely selectively inhibits cellular mRNA splicing that contributes to host shutoff without interfering with viral mRNAs (Figure 1). 

HSV-2 encodes the ICP27 protein that binds directly to pre-mRNAs and prevents their splicing [79]. Unlike HSV-2, the human immunodeficiency virus 1 (HIV-1) Vpr protein binds to the spliceosome protein SAP145 to inhibit host pre-mRNA splicing [80]. Recently, it was found that IAV endoribonuclease PA-X protein targets splicing machinery that leads to preferable degradation of cellular mRNAs with more splicing sites [81,82]. However, the exact molecular mechanism is yet to be fully elucidated. Splicing blocking may lead to mRNA with a premature stop codon or disruption of the ORF, resulting in the production of aberrant proteins that are promptly degraded. 

### 5.4. mRNA Export 

Pre-mRNA processing occurs in the nucleus, after which, the mature mRNA must be exported to the cytoplasm such that translation can occur. Some viruses can target the export of host mRNAs by disrupting the nuclear-cytoplasmic transport machinery. Rhinovirus 2A and 3C proteases cleave vital constituents of nucleocytoplasmic mRNA export machinery [83]. The IAV protein NS1 and VSV matrix protein downregulate and competitively bind the Nup98 protein, respectively, which is required for mRNA export [84,85]. Additionally, IAV’s NS1 protein interacts with and sequesters key mRNA export machinery proteins (p15, E1B-AP5, Rae1, and NXF1) to form an inhibitory complex, thereby inducing mRNA export blockage [85]. During VACV infection, the nuclear pore complex is required for efficient VACV replication [86]; however, whether this involves mRNA transport is not clear and is worthy of investigation. 

## 6. Induction of mRNA Degradation

VACV infection promptly induces host cellular RNA degradation. The mRNAs of cellular housekeeping genes, such as β-actin and α-tubulin mRNAs, are progressively degraded over time during VACV infection, and they are almost entirely degraded by 10 h post-infection [87]. This process is highly efficient considering that the mammalian cellular mRNAs have an average half-life of approximately 6.9 h [88]. Earlier studies from Rice et al. postulated that RNA degradation during VACV infection could occur due to several reasons [87]: (1) the synthesis of viral RNases; (2) rapid turnover of mRNAs due to re-compartmentalization of mRNA in morphologically altered infected cells; and (3) interferon-induced 2′5′-oligoadenylate synthetase (OAS) through the activation of endonuclease RNase L, which does not discriminate between host and viral mRNA. 

Although the aforementioned postulates could be true, subsequent findings have demonstrated that VACV encodes two decapping enzymes (D9R and D10R) that contain the Nudix hydrolases motif, which are likely the major driving force of mRNA degradation in VACV-infected cells [58,59]. These decapping enzymes containing a Nudix motif that hydrolyzes a nucleoside diphosphate linked to any moiety X. Initial reports demonstrated that D9 and D10 negatively regulated gene expression, independent of the promoter used, suggesting they target a post-transcriptional step to decrease gene expression. However, the expression of a gene under an encephalomyocarditis (EMC) virus leader sequence that underwent 5′-cap-independent translation using the internal ribosome entry site (IRES) was not suppressed by D9 or D10 overexpression [89]. Additional findings demonstrated that D9 and D10 lead to accelerated degradation of transcripts that are capped (m^7^GpppN) at the 5′ end, which is a feature of both VACV and host cell mRNAs. In fact, by cleaving the 5′-cap, D9 and D10 render the mRNAs for degradation by the 5′-3′ exonuclease Xrn1 (Figure 1) [60]. The importance of D9 and D10 is further accentuated by the fact that a homolog of D10 is found in all chordopoxviruses, whereas the D9 homolog is found in most chordopoxviruses [58]. We recently showed that the depletion of cellular mRNA is a significant contributor to VACV-induced host protein synthesis shutoff [90]. Degradation of host mRNA leading to host shutoff is vital for VACV replication, as it not only usurps the host innate immune response but also reallocates the translation machinery to viral mRNAs. Interestingly, D9 and D10 likely also induce the rapid degradation of viral mRNAs. How VACV mitigates D9 and D10’s effect to speed up viral mRNA turnover for viral protein synthesis is an interesting area we are actively pursuing. Another related open question is whether D9 and D10 target any specific mRNA population to induce degradation.

The induction of host mRNA degradation is a frequently used strategy by many viruses. The SARS-CoV NSP1 protein, IAV PA-X protein, HSV virion host shutoff (VHS) protein, Epstein-Barr virus (EBV) BGLF5 protein, and KSHV SOX protein have exo- or endoribonuclease activities and decrease global host protein synthesis by decimating host mRNAs [82,91,92,93,94]. Interestingly, most of these ribonucleases could not distinguish between cellular and viral mRNAs. Other mechanisms are needed to cause a profound host shutoff while still keeping efficient viral protein synthesis. 

## 7. Usurping Host mRNA Translation Machinery

### 7.1. An Overview of Eukaryotic mRNA Translation and Diverse Strategies Employed by Different Viruses to Inhibit Host Translation

All viruses rely on their infected host cells for mRNA translation as they do not encode genes for translation machinery. In many cases, viral and cellular mRNA translation represents a significant conflict of interest to compete for translation machinery. Not surprisingly, many viruses target translation processes to gain a translational advantage for viral mRNAs over cellular mRNAs. More efficient utilization of the translation machinery by viral mRNAs would ultimately put host mRNA translation at a disadvantage and contribute to host protein synthesis shutoff. 

Messenger RNA translation is the most energy-consuming process in a cell. Cap-dependent translation is the dominant mode of eukaryotic mRNA translation with three major steps: initiation, elongation, and termination. In eukaryotic cells, the initiation factor 2 (eIF2) forms a ternary complex (TC) with initiator-methionine tRNA (Met-tRNAi) and GTP. Consequently, TC binds to the 40S ribosome in complex with eIF1, eIF3, and eIF5 to form the 43S pre-initiation complex. A rate-limiting translation initiation step is followed by the recruitment of a hetero-trimeric complex called eIF4F on mRNA. The eIF4F complex consists of an m^7^G cap-binding protein eIF4E, RNA helicase eIF4A, and scaffold protein eIF4G. Once formed, the eIF4F complex binds the m^7^G cap of the mRNA, and the scaffold protein eIF4G interacts with the poly(A) binding protein (PABP) bound to the 3′ poly(A) tail to promote transient 5′-3′ communication, which is known as the closed-loop model. Subsequently, by binding to the eIF3 complex, eIF4G helps to recruit the 43S pre-initiation complex on the mRNA to form the 48S pre-initiation complex. The 48S pre-initiation complex then scans the mRNA in the 5′→3′ direction until it reaches the start codon, usually AUG. Once the start codon is recognized, eIF5B mediates the hydrolysis of eIF2-bound GTP. This change prompts the joining of the 60S ribosome to the 48S pre-initiation complex to form an elongation-competent 80S complex [95,96]. This sophisticated process is followed via translation elongation, termination, and ribosome recycling. Since translation initiation is the rate-limiting step of translation, many viruses target different proteins involved in this process. 

The multi-subunit complex eIF4F is a primary target for viruses to hijack host translation. All three proteins in the eIF4F complex are targeted by different viruses belonging to different families. FMDV protease 3C cleaves eIF4A to block host translation [97]. HSV-1 VHS protein binds to eIF4A to gain proximity to cleave and degrade host mRNAs [98]. Enterovirus (EV) 2A, various retrovirus proteases, FMDV leader protease, and feline calicivirus 3C protease cleave eIF4G [99,100,101,102]. Viral proteins, such as rhinovirus 2A and rotavirus NSP3, bind eIF4G and displace PABP to prevent the closed-loop conformation required for translation in many host mRNAs [103,104]. Cap-binding protein eIF4E is a common target for many viruses to reduce host translation. Some viral proteins bind and recruit eIF4E to viral mRNA to gain a translation advantage. Notable examples include the Adenovirus shutoff protein 100K and turnip mosaic virus (TMV) VPg protein, while *Cripavirus* and influenza virus perform this function using unknown proteins [105,106,107,108]. Enteroviruses (EV) use a distinctive approach to target eIF4E, by which virus-induced miR-141 suppresses eIF4E mRNA translation to limit the availability of this protein [109]. Many viruses also target the phosphorylation state of translation repressor protein eIF4E binding protein 1 (4EBP1). 4EBP1 is a translation repressor that limits the availability of eIF4E to form the eIF4F complex [110]. Hyperphosphorylated (four sites) 4EBP1 releases eIF4E for eIF4F complex formation. SV40 small t antigen carries out the PP2A-dependent dephosphorylation of 4EBP1, whereas VSV M protein and reovirus p17 dephosphorylate 4EBP1 through inactivating Akt-mTOR, which is required for 4EBP1 hyperphosphorylation [111,112,113]. In doing so, viruses induce host shutoff since the vast majority of host mRNAs depend on eIF4F-complex-reliant cap-dependent translation. 

Viruses are known to alter the function of multifactor initiation complexes other than eIF4F to induce host shutoff. Alphaviruses (Sindbis and Semliki Forest virus) induce phosphorylation of eIF2α to block global host translation [114]. eIF3 is a multiprotein complex composed of 13 different subunits, several of which are known targets for viruses to decimate host mRNA translation. Measles N protein binds eIF3g, whereas rabies M protein binds eIF3h to impede host translation [115,116]. Similarly, SARS-CoV and infectious bronchitis virus (IBV) spike protein binds eIF3f to impair host mRNA translation [117]. Enteroviruses encode 3C proteases that cleave eIF5B, thus impairing translation by preventing eIF5B from interacting with eIF1A and the ribosome to accurately position met-tRNA on the start codon of an mRNA [118]. Some viruses, such as FMDV, use multiple modes to induce host shutoff. In addition to cleaving eIF4A and eIF4G, FMDV infection induces the cleavage of eIF3a, eIF3b, and PABP [97]. 

PABP binds to the 3′ poly(A) tail of an mRNA to enhance RNA stability, and this interaction is also vital for translation initiation complex formation in the cytoplasm [119]. Many viruses target PABP to induce host shutoff. Calicivirus and enterovirus protease 3C or 3C-like protein cleaves PABP to inhibit its function [120,121]. HIV-1 protease, Rubella capsid protein, and influenza NS1 bind PABP and suppress host translation [122,123,124]. Rotavirus NSP3 displaces PABP from eIF4G and interacts with RoXaN to cause nuclear accumulation of PABP [104,125]. The ORF57 (HSV-1 ICP27 homolog) and K10 proteins of KSHV and HSV-1 UL47 protein bind PABP to cause nuclear accumulation and abolish its function in the cytoplasm [126,127,128].

To efficiently produce viral proteins during the shutoff, many viruses have evolutionarily acquired cis-elements in mRNA to induce selective viral protein synthesis. The IRES is used by many RNA viruses, as well as some DNA viruses. Notable viruses that were suggested to be able to use IRES to mediate translation initiation via cap-independent mode to produce proteins include coronavirus, HCV, CSFV, HIV, and CrPV [129,130,131,132,133]. Influenza B virus induces the combined translation of both M1 and BM2 protein via the base pairing of mRNA with 18S ribosomal RNA to promote the re-initiation of translation [134]. Adenovirus uses ribosome shunting to enhance viral mRNA translation, where the tripartite leader in the non-coding region of viral late mRNAs exhibits high complementarity with 18S rRNA, which promotes the ribosome shunting mechanism [135]. Viruses are notorious for recruiting translation initiation factors to their mRNAs to promote viral mRNA translation. Calicivirus VPg protein binds and recruits eIF3 and eIF4E to viral mRNA [136,137] and HSV-1 ICP27 and UL47 proteins bind PABP [126]. As such, the recruitment of initiation factors to viral mRNAs stimulates their translation. During replication, viruses produce dsRNA that is sensed by innate immune molecule PKR, leading to PKR and downstream eIF2α phosphorylation [138]. When eIF2α is phosphorylated, global inhibition of translation initiation occurs [139]. To avert this situation, viruses use various strategies to hinder PKR-mediated eIF2α-phosphorylation. Influenza NS1 and HCMV’s two related proteins (TRS1 and IRS1) sequester dsRNA and prevent PKR phosphorylation [140,141]. EBV SM and KSHV ORF57 directly sequester PKR to thwart PKR activation [142,143]. HPV E6 and HSV ICP34.5 proteins regulate eIF2α phosphatase to dephosphorylate eiF2α [144,145]. VACV encodes two PKR inhibitors, namely E3L and K3L. E3L binds to dsRNA to prevent PKR dimerization, whereas K3L, with its homology to eIF2α, acts as a pseudo-substrate for PKR. Many great details on how VACV, as well as other poxviruses, relaxes PKR-mediated translation inhibition have been revealed [146,147,148].

### 7.2. Suppression of Host Cell Translation during VACV Infection 

VACV uses multiple tactics to modulate cellular mRNA translation. Our transcriptome-wide analysis showed that VACV mRNAs have a higher translation efficiency than host mRNAs [149]. During VACV infection, prompt inhibition of cellular protein synthesis occurs. Earlier findings have shown that VACV infection results in the inhibition of protein synthesis via a surface tubular element (STE) displayed on the VACV membrane. However, VACV STE did not affect either cellular RNA or DNA synthesis. When the authors exposed cells to purified STE, a decrease in the polyribosome occurred, accompanied by an increase in the free ribosome pool [150]. 

A remarkable factor present in virions implicated in inducing host shutoff is the phosphorylated 11 kDa protein [151], encoded by F17R [152,153]. Purified 11 kDa/F17 protein from VACV virion and cell-free extract from VACV-infected cells prevents methionyl-tRNA_fMet-40S initiation complex formation, thereby inducing host shutoff [151,154]. The importance of this small protein is further augmented by the finding that preventing the expression of F17 protein interrupts VACV morphogenesis [153]. It was recently shown that F17 sequestering of Rictor and Raptor dysregulates mTOR to counter the antiviral response while retaining mTOR-mediated enhancement of viral protein synthesis [152]. These findings indicate that F17 has multiple roles ranging from inducing host shutoff and countering the antiviral response to enhancing viral protein synthesis during infection. 

VACV expresses a protein using its early gene VACWR169, which is confined in the host cell cytoplasm. In the cytoplasm, protein 169 impairs host protein synthesis, thereby facilitating the inhibition of host antiviral responses. Unlike other factors that induce host shutoff, protein 169 targets translation initiation by affecting both cap-dependent and cap-independent mechanisms, although how this protein manages to do it is yet to be elucidated. Protein 169 is not vital to VACV replication and spread in culture cells; however, it is required to regulate virulence as a VACV lacking protein 169 causes severe infections that induce stronger immune responses and are thus promptly cleared. By inducing host protein synthesis shutoff, VACV protein 169 suppresses host antiviral response and hence regulates virulence [155]. Interestingly, full-length 169 is not encoded in all VACV strains; for example, a search of a VACWR169 homolog in the VACV Copenhagen strain reveals a premature stop-codon that would result in a truncated, possibly non-functional, protein product. 

As discussed earlier, VACV-poly(A)-polymerase-induced POLADS may sequester PABP to make them inaccessible for host mRNA translation [69]. In a cell-free protein-synthesizing system, the addition of POLADS inhibits cellular mRNA translation by up to 70%, while viral mRNA displayed minimal inhibition [156,157,158]. The selective host protein synthesis inhibitory property of the POLADS is due to the poly(A) tail, as the increased length of the poly(A) tail in POLADS corresponds to increased inhibitory activity [69,158]. Moreover, the addition of PABP reversed the host cell mRNA translation inhibition by POLADS, suggesting that PABP becomes the limiting factor that may be sequestered by these POLADS for host cell mRNA translation during VACV infection [69,156,158]. These results also suggest that VACV mRNA translation is less dependent on PABP, which is in agreement with the finding that PABP1 is localized outside of viral factories, where viral mRNA translation is concentrated [159,160]. However, the molecular mechanisms involved, as well as whether there is another poly(A)-binding protein that can substitute for PABP, are yet to be discovered. 

Our genome-wide identification of VACV transcription start sites and polyadenylation sites revealed pervasive transcription initiation and termination [161,162]. Most of them are not transcripts of the ≈200 annotated genes. The findings indicate a vast number of “dark” transcripts that are both capped and polyadenylated, which likely include some of the POLADS described above. These transcripts could be competitors of cap and poly(A)-tail-binding proteins necessary for cap-dependent mRNA translation. Because VACV post-replicative mRNA translation is less dependent on these factors, as shown by several groups, including us [149,163,164], these “dark transcripts” can place VACV mRNA at a translational advantage. This hypothesis is under active investigation in our laboratory. 

Recently, antiviral granules (AVGs) and RNA granules were suggested to be present in VACV-infected cells [160,165]. In fact, AVGs contain translation initiation proteins (eIF3h, eIF4E, and PABP), leading to speculation that such redistribution of translation initiation factors could be a mechanism used to limit the availability of these factors for host mRNA translation [160], although further investigation is needed. 

### 7.3. Preferential Translation of VACV mRNAs 

A recent review by Meade et al. very nicely summarized how VACV infection modulates host translation machinery to selectively translate viral mRNAs [166]. We will only give a brief overview of the strategies used by VACV to preferentially translate VACV mRNAs. 

Several studies showed that VACV’s post-replicative mRNA translation is enriched in or near virus replication sites called “viral factories," which are characterized by the intense staining of VACV viral DNA in the cytoplasm [159,167]. It has been shown that translation initiation factors, such as eIF4E and eIF4G, could be recruited to the viral factories, likely enhancing cap-dependent translation initiation [168]. Another study suggests that translation outside of the viral factories could also occur [169]. VACV infection stimulates eIF4F complex formation. The eIF4E is repressed by hypo-phosphorylated eIF4E-binding proteins (4EBPs) [170]. In the early stages of infection, VACV induces surface integrin-β1-mediated PI3K activation, leading to hyperphosphorylation of 4EBP1 and the subsequent release of cap-binding protein eIF4E [171,172], which consequently augments the formation of the eIF4F complex enhancing VACV protein synthesis [172]. Another poxvirus, namely myxoma virus (MYXV), activates AKT using the host range protein MT-5 [173,174], although the role of AKT activation on mRNA translation during MYXV infection has not been studied yet. Additionally, VACV infection activates the MAPK/ERK pathway that stimulates the phosphorylation of eIF4E by MNK1 [159]. Phosphorylation of eIF4E at the serine 209 residue may lead to an increase in translation initiation of VACV mRNAs [175,176,177]. 

The poly(A) leader at the 5′ end of transcripts is a unique feature of all VACV post-replicative mRNAs, which was discovered three decades ago. Only recently, we and others found that the 5′-poly(A) leader confers a selective translational advantage to viral post-replicative mRNAs, specifically in poxvirus-infected cells [149,178,179]. Although the mechanism is still largely unknown, it was suggested that post-translational phosphorylation of small ribosomal protein RACK1 by VACV kinase B1 is necessary for the poly(A)-leader-mediated translational advantage [179]. We found that the poly(A)-headed mRNAs can be efficiently translated in cells with impaired cap-dependent translation, suggesting that it is a cap-independent translation-enhancing element [149]. Moreover, the 5′-poly(A) leader is not an IRES [149]. An A-tract, omega prime, found in the tobacco mosaic virus (TMV) 5′ untranslated region, could also enhance translation [180,181]. The TMV omega prime enhances translation via promoting the recruitment of the eIF4F complex [149,182]. Recently, it was found that mRNAs of yeast-virus-like elements contain a similar non-templated 5′-poly(A) leader, which also drives eIF4E-independent translation [183]. It is of note that these VACV post-replicative mRNAs have 5′-caps [184,185]. As discussed above, VACV modulates cap-dependent translation initiation factors and recruits them to viral replication sites for efficient viral mRNA translation. How the cap-independent translation element of the 5′-poly(A) leader coordinates with cap-dependent translation promotion to facilitate selective translation of viral mRNAs is an active area of investigation in our laboratory. We hypothesize that VACV can utilize the advantages of both cap-dependent and cap-independent translation modes to achieve a maximal translational advantage for viral mRNAs, which is facilitated by viral and cellular factors.

## 8. Selective Synthesis of Viral and Cellular Proteins during VACV-Induced Host Shutoff

Virus-induced global host shutoff is beneficial for viruses since it helps evade the host antiviral immune response. Additionally, shutoff also leads to the reapportioning of cellular machinery and critical host processes to confer a replication advantage to the virus. Although shutoff provides advantages to the virus regarding immune evasion and the reallocation of cellular resources, the selective synthesis of crucial cellular proteins and viral proteins is necessary to sustain virus replication. During VACV-induced host shutoff, viral and specific cellular proteins are selectively synthesized to drive efficient viral replication. The viral post-replicative mRNAs use an evolutionarily optimized 5′-poly(A) leader at the 5′-UTR region to mediate the translation advantage to VACV post-replicative mRNAs. However, during VACV infection, viral proteins augment the translational initiation to provide a translational advantage to the 5′-poly(A) leader bearing the VACV post-replicative mRNAs. Cellular proteins crucial for VACV replication are those of different complexes of oxidative phosphorylation (OXPHOS). The mRNAs encoding for proteins of OXPHOS have a shorter and less complex secondary structure bearing 5′-UTR, which provides a higher translation efficiency during VACV infection. These findings advanced our understanding of selectively synthesized proteins during poxvirus-induced host shutoff. We previously reviewed this topic in more detail [186].

## 9. Induction of Systematic Host Protein Degradation

In addition to manipulating host gene expression at both the DNA and RNA levels, VACV also targets cellular proteins for degradation. A properly functioning ubiquitin-proteasome system has previously been shown to be necessary for viral replication. VACV infection in the presence of proteasome inhibitors significantly reduces the expression of post-replicative genes and fails to produce virus factories, but viral titers can be rescued after treatment removal [187,188,189].

Using a tandem-mass tag (TMT) labeling method, Soday et al. were recently able to determine that 265 host proteins are degraded during VACV infection, including histone deacetylase 5 (HDAC5) by VACV early protein C6, interferon-induced proteins with tetratricopeptide repeats (IFITs), tripartite motif (TRIM) proteins, and cell surface collagens, about 70% of which were determined to be targeted by the 26S proteasome via rescue with the proteasome inhibitor MG132 [3]. Histone deacetylases have previously been shown to play a role as a restriction factor in other viruses, including EBV, HCMV, and HPV, among others [190,191,192], C6 has been shown to inhibit interferon regulatory factors (IRF) 3 and 7 by interacting with proteins of the TANK binding complex (TBK1) [193], as well as being responsible for the proteasomal degradation of HDAC4 [194]. Liu et al. also found that IFITs are targeted for degradation by a VACV ankyrin-repeat/F-box protein C9 [4]. The degradation likely helps VACV evade antiviral responses since IFITs and TRIMs are important players in a host’s defense against viruses [195,196]. There is still much work to be done to determine the VACV proteins responsible for the proteasomal degradation of the many other proteins identified in the above study.

## 10. Conclusions and Perspectives

During infection, VACV makes host cells conducive to viral replication through multiple measures, including inhibiting host cell death, altering cellular metabolism, and causing host shutoff, which is marked by global inhibition of host protein synthesis. To induce host shutoff, VACV employs wide-ranging strategies that target the key processes of gene expression, as well as pre- and post-gene expression processes: inhibiting host DNA synthesis and mRNA transcription, interfering with host mRNA processing and maturation, hijacking host translation machinery to preferentially synthesize viral proteins, and systematically promoting host protein degradation. A combination of these strategies leads to an expeditious, profound, and sustained host shutoff during VACV infection. Accelerated mRNA degradation and inhibition of cellular mRNA translation likely play a key role in the rapid induction of host shutoff, while host mRNA transcription and processing interference, cellular protein degradation, and cellular DNA synthesis inhibition can make the shutoff more profound and lasting during infection. Next, we briefly discuss several topics that we believe are important in this area, but of course, these are not exhaustive.

For most of the tactics VACV employs to induce host shutoff, the factors involved and their underlying molecular mechanisms are still largely unknown. What are the viral factors and how do these factors inhibit cellular DNA replication and transcription? Does VACV interfere with mRNA export? Likewise, how does VACV promote viral mRNA translation, and stimulate host protein degradation? What are the cellular mechanisms exploited by these viral factors? For those processes with identified viral factors responsible—for example, viral decapping enzymes that cause accelerated mRNA degradation, VACV 169 that causes translational repression, the poly(A) leader of viral mRNAs that confers translational advantage—the molecular mechanisms are largely elusive. How these different tactics cooperate during VACV replication to achieve the profound host shutoff is also a fruitful area for exploration. Additionally, while we know a great deal about VACV-induced host-shut shutoff, not much has been revealed in other poxviruses. It is conceivable that many of the strategies used by VACV are also used by other poxviruses. However, we may not assume all processes in VACV infection also occur in other poxvirus infections. In fact, it is of great interest to explore whether other poxviruses have evolved different genes/strategies to fulfill the mission of shutting off the host protein synthesis. Supporting this area of investigations, different poxviruses have evolutionarily acquired a broad range of different genes to address various cellular environments they face.

The viral decapping enzymes (D9 and D10)-induced mRNA degradation is probably the most well-studied host shutoff induction mechanism, with many papers published on this topic. However, with a greater understanding of this process, more intriguing questions arise that demand answers. The knockout of individual decapping enzymes or both enzymes presents various effects on VACV replication in cultured cells and infected animals [197,198,199]. Interestingly, overexpression of these decapping enzymes also causes a decrease in VACV replication [89]. A compelling question is how these decapping enzymes are regulated and coordinated to keep the balance for efficient viral replication. Our unpublished data indicate that the decapping enzymes are also necessary for highly efficient viral mRNA translation during infection. We are actively investigating the mechanism involved in this aspect, as well as the relationship between the induction of mRNA decay and stimulation of the mRNA translation. Another intriguing aspect is whether the decapping enzymes have any selectivity in inducing mRNA decay during infection, as previous studies showed that they could induce both cellular and viral mRNA decay. Supporting this possibility, in vitro studies have shown that D9 decapping activity is more susceptible to RNA inhibition, while D10 is more susceptible to an m^7^G cap inhibition [58,59].

The 5′-poly(A) leader bearing post replicative mRNAs are selectively translated during poxvirus-induced host shutoff. However, the exact mechanism and the host/viral factors involved in this process are still elusive. As mentioned above, we are investigating how the decapping enzymes may stimulate the translation of mRNAs with a poly(A) leader. Other outstanding questions include: Are there specific cellular factors facilitating 5′-poly(A)-mediated translation? Does the 5′-poly(A) leader have the intrinsic property of recruiting ribosomes with low (or no) requirement of eukaryotic translation initiation factors? Does the poly(A) leader facilitate viral mRNA escape from decapping enzyme-induced mRNA decay? It has been shown that the first A of the 5′-poly(A) leaders during VACV infection is N6-methyladenosine (m6A)-modified. As the m6A modification has been suggested in various aspects of mRNA degradation and mRNA translation, it is important to evaluate whether this modification facilitates a viral mRNA advantage during VACV-induced host shutoff. In fact, there is increased stability of m^6^Am RNAs due to resistance to the Dcp2 decapping enzyme in cells [200].

It has been over half of a century since the first few studies described the phenomena of host shutoff upon VACV infection [201,202,203]. Since then, VACV has kept amazing us by revealing how many means it can deploy to achieve this goal. Studies on this subject have revealed several valuable molecular tools produced by VACV, for example, the virus-encoded decapping enzymes, mRNA translation modulators, and the 5′-poly(A) leaders on viral post-replicative mRNAs. However, many outstanding questions deserve further inquiries, which would not only facilitate understanding poxvirus replication but also provide invaluable tools to dissect many aspects of gene expression regulation and cell biology.
